# First-line induction chemotherapy with high-dose methotrexate versus teniposide in patients with newly diagnosed primary central nervous system lymphoma: a retrospective, multicenter cohort study

**DOI:** 10.1186/s12885-023-11268-5

**Published:** 2023-08-11

**Authors:** Kaili Zhong, Yanyan Shi, Yuhuan Gao, Huilai Zhang, Mingzhi Zhang, Qiaohua Zhang, Xinan Cen, Mei Xue, Yan Qin, Yu Zhao, Liling Zhang, Rong Liang, Ningju Wang, Yan Xie, Yu Yang, Aichun Liu, Huizheng Bao, Jingwen Wang, Baoping Cao, Wei Zhang, Weijing Zhang

**Affiliations:** 1 Department of Lymphoma, Beijing Shijitan Hospital, Capital Medical University, Beijing, China; 2 Research Center of Clinical Epidemiology, Peking University Third Hospital, Beijing, China; 3 Department of Hematology, Fourth Hospital of Hebei Medical University (Tumor Hospital of Hebei Province), Shijiazhuang, China; 4 Department of Lymphoma, Tianjin Medical University Cancer Institute and Hospital, Tianjin, China; 5 Department of Oncology, The First Affiliated Hospital of Zhengzhou University, Zhengzhou, China; 6 Department of Oncology, Shanxi Academy of Medical Sciences & Shanxi Bethune Hospital, Shanxi Bethune Hospital affiliated to Shanxi Medical University, Taiyuan, China; 7 Department of Hematology, Peking University First Hospital, Beijing, China; 8 Department of Hematology, Air Force Medical Center, Beijing, China; 9 Department of Medical Oncology, National Cancer Center/National Clinical Research Center for Cancer/Cancer Hospital, Chinese Academy of Medical Sciences & Peking Union Medical College, Beijing, China; 10 Department of Hematology, the General Hospital of PLA, Beijing, China; 11 Department of Lymphoma, Cancer Center, Union Hospital, Tongji Medical College, Huazhong University of Science and Technology, Wuhan, China; 12 Department of Hematology, Xijing Hospital, Air Force Military Medical University, Xi’an, China; 13 Department of Medical Oncology, General Hospital of Ningxia Medical University, Yinchuan, China; 14 Departments of Lymphoma, Key Laboratory of Carcinogenesis and Translational Research, Peking University Cancer Hospital and Institute, Beijing, China; 15 Department of Medical Oncology, Fujian Cancer Hospital & Fujian Medical University Cancer Hospital, Fuzhou, China; 16 Department of Hematology and Lymphoma, Cancer hospital of Harbin Medical University, Haerbin, China; 17 Department of Medical Oncology, Jilin Cancer Hospital, Changchun, China; 18 Department of Hematology, Beijing Tongren Hospital, Capital Medical University, Beijing, China; 19 Department of Hematology, Peking Union Medical College Hospital, Beijing, China

**Keywords:** First-line induction chemotherapy, Methotrexate, Primary central nervous system lymphoma, Teniposide

## Abstract

**Background:**

This study aimed to compare the efficacy and safety of high-dose methotrexate (HD-MTX) versus teniposide (TEN) in patients with newly diagnosed immunocompetent primary central nervous system lymphomas (PCNSLs).

**Methods:**

The study included immunocompetent, adult patients with newly diagnosed PCNSL at 22 centers in China from 2007 to 2016. The patients received HD-MTX or TEN as first-line induction therapy. The objective response rate, progression-free survival, and overall survival were analyzed for each patient cohort.

**Results:**

A total of 96 patients were eligible: 62 received HD-MTX, while 34 received teniposide. The overall response rate was 73.2% and 72.7% in the MTX and the TEN cohorts, respectively (*P* = 0.627). The median progression-free survival was 28.4 months [95% confidence interval (CI): 13.7–51.2] in the MTX cohort and 24.3 months (95% CI: 16.6–32.1) in the TEN cohort (*P* = 0.75). The median overall survival was 31 months (95% CI: 26.8–35.2) in the MTX cohort and 32 months (95% CI: 27.6–36.4) in the TEN cohort (*P* = 0.77). The incidence of any grade of coagulopathy/deep-vein thrombosis and gastrointestinal disorders was significantly higher in the MTX cohort than in the TEN cohort; no significant difference was found in the incidence of other adverse events between the two cohorts.

**Conclusions:**

This was the first multicenter study using TEN as the main agent compared with HD-MTX in newly diagnosed primary CNS lymphoma. The TEN-based regimen was non-inferior to the HD-MTX-based regimen with similar overall responses.

**Classification of evidence:**

This study provided Class III evidence that the teniposide-based regimen was non-inferior to high-dose methotrexate − based regimen with similar overall responses and long-time survival in immunocompetent patients with PCNSL.

## Background

Primary central nervous system lymphoma (PCNSL) accounts for 2%–3% of all non-Hodgkin’s lymphoma cases with increasing incidence, particularly for elderly patients [1]. The standard therapeutic regimens for newly diagnosed PCNSL have not been well defined. Most regimens of induction chemotherapy include drugs that cross the blood–brain barrier (BBB) in conventional doses. High-dose methotrexate (HD-MTX)-based regimen is now considered the standard treatment regimen [2]. However, HD-MTX treatment − associated overall survival (OS) was less than 20%–35% [3, 4]. Even combined with cytarabine, temozolomide, etoposide, the 5-year OS rate (30% -50%) of HD-MTX based scheme is still unsatisfactory [2, 5–8] and more severe hematological toxicity. For these reasons, the development of more effective and less toxic novel therapeutic regimens for PCNSL is of great clinical importance.

High doses of intravenous methotrexate are necessary (≥ 3.5 g/m^2^) to overcome the BBB [5, 9–12], but the problem associated with it is the toxicity it causes. The median age at diagnosis of PCNSL is 60–65 years in China. Among them, a significant portion of elderly patients aged 70 or older cannot tolerate HD-MTX, and other equivalent drugs or treatment options for those patients are currently lacking. Many water-soluble drugs have been proven ineffective in PCNSL because of their poor ability to penetrate the BBB [13]. Teniposide, as a highly fat-soluble topoisomerase II–trapping agent, is a potent and broad-spectrum antitumor drug [14–17]. Teniposide showed anti-lymphoma ability together with other drugs, such as methotrexate, fotemustine, and dexamethasone in a small number of cases [15, 18, 19]. In a comparative study, researchers analyzed the efficacy and safety of flumustine, teniposide, and dexamethasone in patients with central nervous system lymphoma (CNSL). The results showed that 8 patients with PCNSL had an overall response rate of 88%, whereas 8 patients with secondary CNSL had an overall response rate of 63%. This study suggested that a teniposide-based regimen might be an effective treatment for CNSL [11]. A phase II clinical trial found high response rates in patients with PCNSL treated with MTX, teniposide, carmustine, and methylprednisolone after radiotherapy [14]. A clinical study in China found that, compared with HD-MTX alone, HD-MTX combined with teniposide was more effective in treating PCNSL and could effectively control tumor progression, prolong survival, and improve prognosis [15]. An early clinical study at our center confirmed that teniposide with or without rituximab was well tolerated and effective in patients with PCNSL, with a high response rate and promising long-term survival [20]. Although the efficacy of teniposide-based regimens in patients with PCNSL has been demonstrated, the efficacy and safety of teniposide versus HD-MTX have not been reported.

In this retrospective multicenter study, we compared the efficacy and safety of TEN and HD-MTX with or without rituximab as first-line chemotherapy in patients with newly diagnosed PCNSL. We aimed to provide a new regimen of simple drugs as a candidate choice other than HD-MTX.

## Methods

### Study design and patients

This was a retrospective multicenter study of immunocompetent adults with newly diagnosed PCNSL at 22 centers in China from 2007 to 2016. The inclusion criteria were as follows: patients ≥ 18 years older; immunocompetent patients; patients with newly diagnosed PCNSL confirmed by histology or cytology from cerebrospinal fluid (CSF); patients with no previous cytotoxic treatment; patients with no evidence of extra-CNS involvement; patients receiving either high-dose methotrexate (HD-MTX) (MTX cohort: high-dose methotrexate-based regimen treatment, 3–3.5 g/m^2^ methotrexate daily on day 1 every 14 days, with or without rituximab 375 mg/m^2^ on day 0) or teniposide (TEN cohort: teniposide-based regimen treatment, 60 mg/m^2^ teniposide daily on days 1–5 every 21 days, with or without rituximab 375 mg/m^2^ on day 0) as the first-line induction therapy. The major exclusion criteria were concomitant immunosuppression, including positive human immunodeficiency virus serology; no assessment of chemotherapy, and no long-term survival result. All clinical management decisions and response evaluations were independently performed by the patients’ treating physicians. The study was approved by the local institutional ethic committee. As our study involved only a retrospective review of previous clinical data, the requirement for informed consent was waived.

The demographic data and clinical features of all patients were obtained retrospectively from the medical records. The clinical variables included age at diagnosis, sex, ethnicity, height, weight, body surface area, Eastern Cooperative Oncology Group (ECOG) performance score, year of diagnosis, symptoms at diagnosis, presence/absence of B symptoms, result of pathology, International Extranodal Lymphoma Study Group (IELSG) score of primary CNS lymphoma, lactate dehydrogenase (LDH), protein level of CSF, sites of intracranial lesions, comorbidities (hypertension, coronary disease, diabetes, hyperlipidemia, viral hepatitis B, etc.), induction regimen and its toxicity, inclusion of rituximab, number of cycles, response (complete response, CR; partial response, PR; stable disease, SD; progressive disease, PD), use/type/timing of stem cell transplantation, use of radiotherapy, progression-free survival (PFS), OS, and cause of death.

The purpose of this study was to compare the efficacy and safety of a teniposide-based regimen with a high-dose methotrexate-based regimen in patients with newly diagnosed primary CNS lymphoma. The outcomes were objective response rate [including complete response rate and overall response rate (ORR) after induction chemo/immunotherapy], PFS, and OS. The OS was calculated from the time of diagnosis to death of any cause or, for the patients alive, to the date of the last follow-up, whereas PFS was the time from diagnosis to the time of relapse or progression or death from any cause. The International PCNSL Collaborative Group Response Criteria [21] was used for response assessment. Our study also analyzed the toxicity of patients in these 2 cohorts 1 month after chemotherapy. The toxicities of MTX and TEN cohorts were assessed with the Common Terminology Criteria for Adverse Events version 4.0.

### Statistical analysis

The baseline (pretreatment) and treatment variables were collected, along with dates of first progression, last follow-up, and death. The normally distributed variables were expressed as mean ± standard deviation (SD), and the skewed or unknown distributed variables were displayed as the median and interquartile range (IQR). The categorical variables were presented as count (percentage). The univariate analyses (UVA) for OS were performed using each of the pretreatment variables evaluated. The survival analyses were performed regardless of the duration or type of therapy received. PFS and OS rates were estimated using the Kaplan–Meier method, and differences were assessed with the log-rank (Mantel–Cox) test. Variables with a *P* value < 0.05 on UVA were included in the stepwise multivariate Cox proportional-hazards model. Hazard ratios (HRs) and their 95% confidence intervals (CIs) were calculated. The Cox proportional-hazards model was recalculated with the addition of an induction regimen as a variable to evaluate the impact of the induction regimen. Kaplan–Meier survival curves were generated. Analyses were done with IBM SPSS software (version 20·0). The differences in categorical data were calculated using the chi-square or Fisher’s exact test with significance defined as *P* ≤ 0.05. All *P* values were two tailed.

## Results

### Comparison of clinical characteristics of patients in HD-MTX and teniposide cohorts

Full survival data were available for 96 eligible patients from 22 centers in China. Of these, 62 patients received treatment with MTX monotherapy with or without rituximab (MTX cohort), and 34 patients received teniposide monotherapy with or without rituximab (TEN cohort). The proportion of patients whose tissue samples were obtained by stereotactic biopsy in the MTX cohort was significantly lower than that in the TEN cohort (29.5% vs 50.0%,), while the proportion of patients whose tissue samples were obtained by surgical partial resection was significantly higher than that in the TEN cohort (70.5% vs 50.0%) (*P* = 0.047). No significant differences were found in age, sex, ECOG performance status, IELSG risk, LDH level, CSF cytology, lymphoma categories, comorbidities, and the combination of MTX/TEN with rituximab and radiotherapy between the 2 cohorts (Table 1). The median age of the whole cohort was 55 years (range, 22–74 years), with 51.5 years in the TEN cohort (range, 22–74 years) and 58 years in the MTX cohort (range, 23–74 years) (*P* = 0.655). The ratio was 1.13:1 in all patients, with no difference between the two cohorts. Nearly half of the patients had poor performance status (ECOG PS ≥ 2). More than 95% patients in both cohorts had B-cell lymphoma, and most of them had diffuse large B-cell lymphoma. Most patients were diagnosed by stereotactic or surgical biopsy, and only one patient in the MTX cohort was diagnosed by cytology from CSF. The comorbidities in two groups were similar, including hypertension, coronary disease, diabetes, hyperlipidemia, viral hepatitis B. In MTX group, there was one patient with depressive state, one patient cured gastric cancer, and one patient with hysterectomy. In TEN group, there was 1 patient with a mass on kidney without pathological diagnosis and 1 patient with hysterectomy. Less than half of the patients in both cohorts received MTX or teniposide with rituximab as the first-line induction therapy, and nearly half of the patients received radiotherapy as a consolidation therapy after MTX or teniposide.

**Table 1:** Patients’ characteristics and distribution of lymphoma categories in the two treatment cohorts

	MTX (*n* = 62)	Teniposide (*n* = 34)	*P* value
*Age (year)*			0.655
< 60	41 (66.1%)	24 (70.6%)	
≥ 60	21 (33.9%)	10 (29.4%)	
*Sex*			0.688
Male	32 (51.6%)	19 (55.9%)	
Female	30 (48.4%)	15 (44.1%)	
*ECOG performance status*			0.657
0–1	33 (54.1%)	20 (58.8%)	
2–4	28 (45.9%)	14 (41.2%)	
*IELSG risk*			0.619
0–1 (low)	18 (32.1%)	11 (33.3%)	
2–3 (intermediate)	31 (55.4%)	20 (60.6%)	
4–5 (high)	7 (12.5%)	2 (6.1%)	
LDH (U/L)			0.127
Normal	49 (79%)	31 (91.2%)	
Increased	13 (21%)	3 (8.8%)	
*CSF cytology*			0.815
Negative	61 (98.4%)	34 (100%)	
Positive	1 (1.6%)	0	
*Tissue sample obtained by*^*a*^			0.047
Stereotactic biopsy	18 (29.5%)	17 (50.0%)	
Surgical partial resection	43 (70.5%)	17 (50.0%)	
*Lymphoma categories*			0.376
Diffuse large B-cell lymphoma	59 (95.2%)	32 (96.8%)	
Burkitt/Burkitt-like lymphoma	0	1 (1.6%)	
Small B-cell lymphoma	1 (1.6%)	0	
Anaplastic large-cell lymphoma	1 (1.6%)	0	
T lymphoblastic lymphoma	1 (1.6%)	0	
T-cell lymphoma, unclassified	0	1 (1.6%)	
*Comorbidities*	19 (30.6%)	12 (35.3%)	0.806
*MTX/TEN combination*			0.501
With rituximab	23 (37.1%)	15 (44.1%)	
Without rituximab	39 (62.9%)	19 (55.9%)	
*Radiotherapy*			0.161
Yes	25 (42.4)	19 (57.6)	
No	34 (57.6)	14 (42.4)	

Data are expressed as *n* (%)

^a^Tissue sample for diagnosis was obtained by CSF cytology examination only in one patient in the MTX cohort

### Comparison of response rates in patients treated with MTX and TEN

The response rate was 90.3% in the MTX cohort and 97.1% in the TEN cohort. The median follow-up duration was 44 months (range, 1–81 months) in the MTX cohort and 39 months (range, 2–61 months) in the TEN cohort. The ORR was 73.2% and 72.7% in the MTX and TEN cohorts (*P* = 0.627). The complete response rate in the MTX cohort was higher than that in the TEN cohort, with no statistically significant difference (*P* = 0.182). According to the IELSG score, no difference in overall responses was found among three levels (*P* = 0.467). Four patients in the MTX cohort and 1 patient in the TEN cohort received autologous hematopoietic stem cell transplantation (ASCT) after the methotrexate and teniposide induction therapy. It might partially explain the higher complete response rate in the MTX cohort. None of the enrolled patients underwent allogeneic hematopoietic stem cell transplantation. Of all 89 patients who received at least 1 dose of first-line chemotherapy and fulfilled all eligibility criteria, 2 (3.6%) died during first-line therapy in the MTX cohort. The causes of death were cerebral bleeding (*n* = 1, 1.8%) and acute toxic effect (*n* = 1, 1.8%) (Table 2).

**Table 2:** Response rates by induction therapy in both treatment cohorts

	MTX (*n* = 56)	Teniposide (*n* = 33)	*P* value
Complete response^a^	28 (50%)	13 (39.4%)	0.182
Partial response	13 (23.2%)	11 (33.3%)	
Stable disease	4 (7.1%)	5 (15.2%)	
Progressive disease	9 (16.1%)	4 (12.1%)	
Died on therapy	2 (3.6%)	0	
Overall response	41 (73.2%)	24 (72.7%)	0.627
*ORR/IELSG score*			
Low risk	12/18 (66.7%)	8/11 (72.7)	
Intermediate risk	24/31 (77.4%)	14/20 (70%)	0.467
High risk	5/7 (71.4%)	2/2 (100%)	

The seven patients who could not be evaluated for response had insufficient imaging data or available medical records. However, some of these patients could be included in the survival analysis. Data are expressed as *n* (%) or *n*/*N* (%) unless otherwise stated

^a^Complete response rate and overall response rate (ORR) for both cohorts according to the International Extranodal Lymphoma Study Group (IELSG) risk score[22]. No interaction between treatment cohort and IELSG risk score was detected (*P* = 0.655)

### Comparison of PFS and OS in the MTX and TEN cohorts

**Fig. 1:**
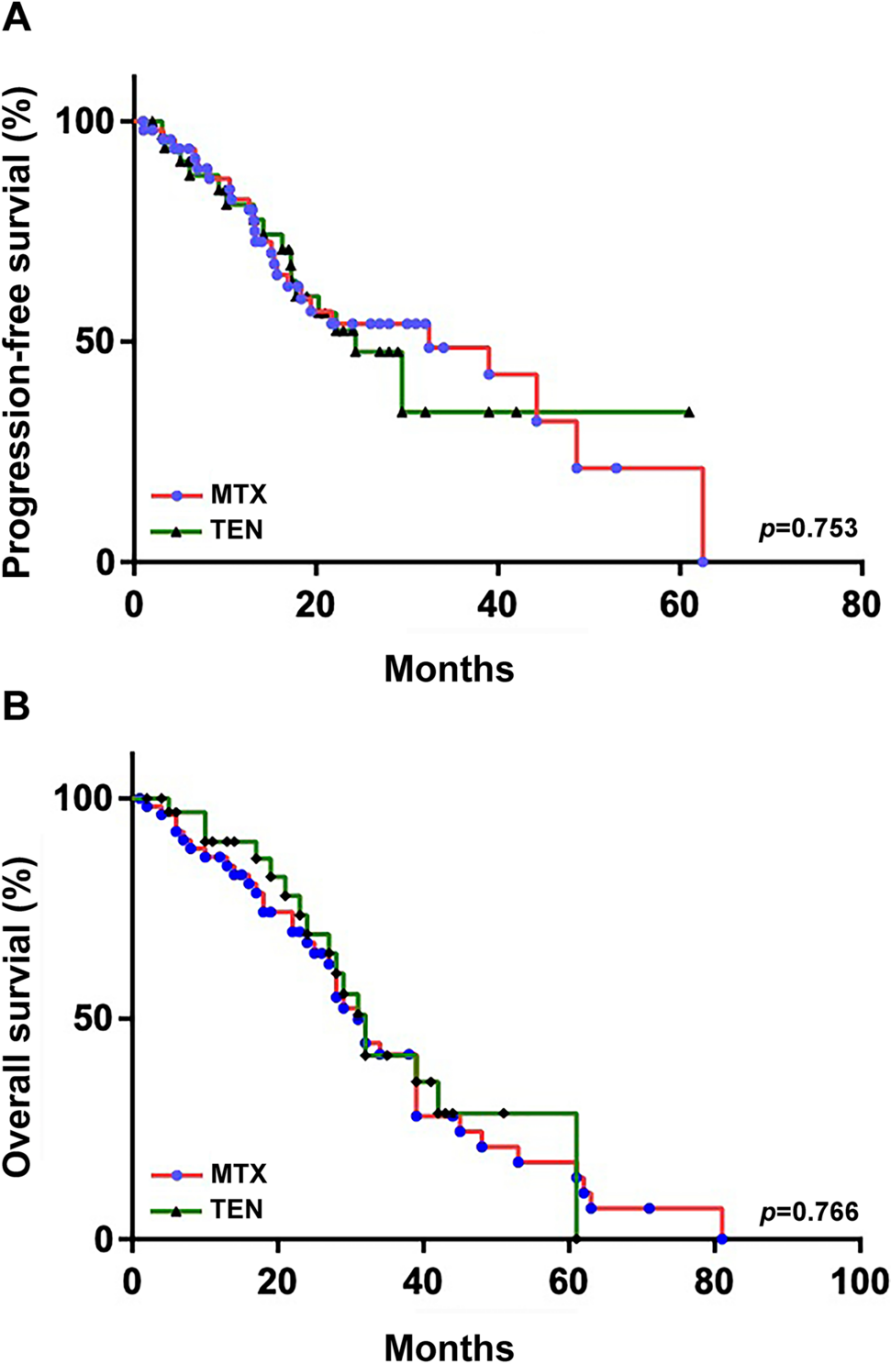
Comparison of progression-free survival and overall survival in the two cohorts. Progression-free (**A**) and overall (**B**) survival of patients treated with HD-MTX or teniposide. MTX, high-dose methotrexate; TEN, teniposide

During follow-up, the median PFS was 28.4 months (95% CI: 13.7–51.2) in the MTX cohort and 24.3 months (95% CI: 16.6–32.1) in the TEN cohort (*P* = 0.75; Fig. 1A), with a 3-year PFS of 48.7% and 34.1%. The median OS was 31 months (95% CI: 26.8–35.2) in the MTX cohort and 32 months (95% CI: 27.6–36.4) in the TEN cohort (*P* = 0.77; Fig. 1B), with a 3-year OS of 41.9% and 41.7%, respectively. A total of 42 events were reported in the MTX cohort (relapse after responsive or stable disease in 25 patients, death while relapse-free in 2 patients, death while early progressive disease in 2 patients, death due to tumor progression in 12 patients, and death for unknown reason in 1 patient), and 33 events in the TEN cohort (relapse after responsive or stable disease in 17 patients, death while relapse-free in 2 patients, death while early progressive disease in 1 patient, death due to tumor in 13 patients, and death for unknown reason in 1 patient).

### Cox regression analysis of prognostic factors for PFS and OS

The univariate analysis found that age, ECOG, IELSG risk, rituximab, and radiotherapy were not significantly associated with PFS. The multivariate analysis found radiotherapy was significantly associated with PFS (HR 0.40, 95% CI, 0.20–0.80, *P* = 0.009).

The univariate and multivariate analyses found that age, ECOG, rituximab, and radiotherapy were not significantly associated with OS. However, the IELSG risk was significantly associated with a worse OS in univariate and multivariate analyses (low vs intermediate, *P* = 0.003; low vs high, *P* = 0.002) (Table 3).

**Table 3:** Univariate and multivariate analyses for progression-free survival and overall survival

	**Univariate analysis (simple Cox regression model)**		**Multivariate analysis (multiple Cox regression model without variable selection)**	
	Hazard ratio (95% CI)	*P* value	Hazard ratio (95% CI)	*P* value
**Progression-free survival**				
**Age**^**a**^	1.039 (0.441–2.449)	0.930	1.675 (0.630–4.457)	0.301
**ECOG**^**b**^	1.729 (0.967–3.091)	0.065	1.324 (0.609–2.879)	0.479
**IELSG risk**				
**Low vs intermediate**	1.983 (0.992–3.965)	0.053	2.384 (0.887–6.409)	0.085
**Low vs high**	2.716 (0.929–7.939)	0.068	1.867 (0.376–9.269)	0.445
**With rituximab**^**c**^	0.949 (0.533–1.690)	0.860	0.766 (0.373–1.576)	0.470
**With radiotherapy**^**d**^	0.468 (0.261–0.838)	0.110	0.404 (0.204–0.800)	0.009
**Overall survival**				
**Age**	1.502 (0.574–3.933)	0.407	2.079 (0.650–6.651)	0.217
**ECOG**	2.036 (0.995–4.165)	0.052	1.049 (0.414–2.659)	0.920
**IELSG risk**				
**Intermediate vs low**	4.992 (1.723–14.458)	0.003	6.079( 1.665–22.200)	0.006
**High vs Low**	8.228 (2.116–31.994)	0.002	6.879 (1.045–45.278)	0.045
**With rituximab**	1.267 (0.643–2.497)	0.493	0.871 (0.370–2.049)	0.752
**With radiotherapy**	0.577 (0.294–1.134)	0.110	0.466 (0.211–1.029)	0.058

^a^Age of 60 years or older versus younger than 60 years

^†^ECOG performance status 0–1 versus 2–4

^c^First-line chemotherapy (methotrexate or teniposide) with or without rituximab

^d^With or without radiotherapy as a consolidation therapy after methotrexate or teniposide

### Comparing the incidence of adverse events in the MTX and TEN cohorts

Table 4 summarizes all hematological and non-hematological toxic effects that occurred during first-line chemotherapy. The prevalence of hematological toxic effects in the two cohorts was similar. The coagulopathy or DVT was more common in the MTX cohort: 13.5% with grade 1 and 1.9% with grade 5 in the MTX cohort versus 0 in the TEN cohort. The gastrointestinal toxic effects increased in the TEN cohort, but all were of grade 1–2. Acute neurotoxicity was reported in two patients (1 grade 4 and 1 grade 5, 3.8%) in the MTX cohort compared with one patient (grade 1–2, 3%) in the TEN cohort (*P* = 0.832).

**Table 4:** Toxic effects in each cohort

	Methotrexate (*n* = 52)				Teniposide (*n* = 33)				*P* value
	Grade 1–2	Grade 3	Grade 4	Grade 5	Grade 1–2	Grade 3	Grade 4	Grade 5	
Neutropenia	12 (23.1%)	5 (9.6%)	10 (19.2)	0	6 (18.2%)	6 (18.2%)	8 (24.2%)	0	0.325
Thrombocytopenia	3 (5.8%)	3 (5.8%)	7 (13.5%)	0	5 (15.2%)	0	3 (9.1%)	0	0.793
Anemia	14 (26.9%)	0	1 (19.2%)	0	9 (27.3%)	0	1 (3%)	0	0.869
Febrile neutropenia/infections	12 (23.1%)	2 (3.8%)	1 (1.9%)	1 (1.9%)	7 (21.2%)	1 (3%)	2 (6.1%)	1 (3%)	0.716
Hepatotoxicity	12 (23.1%)	2 (3.8%)	0	0	10 (30%)	2 (6.1%)	0	0	0.355
Nephrotoxicity	4 (7.7%)	0	0	0	1 (3%)	0	0	0	0.382
Coagulopathy/DVT	7 (13.5%)	0	0	1 (1.9%)	0	0	0	0	0.02
Gastrointestinal	0	1 (1.9%)	0	0	6 (18.2%)	0	0	0	0.01
Mucositis	6 (11.5%)	1 (1.9%)	1 (1.9%)	0	1 (3%)	0	0	0	0.07
Acute neurotoxicity	0	0	1 (1.9%)	1 (1.9%)	1 (3%)	0	0	0	0.832

The worst toxicity per organ, per patient, was considered for analyses. DVT, Deep-vein thrombosis (including pulmonary embolism)

The most common adverse reactions during treatment in patients in the MTX and TEN cohorts included neutropenia, thrombocytopenia, anemia, febrile neutropenia/infections, hepatotoxicity, nephrotoxicity, coagulopathy/DVT, gastrointestinal, mucositis, and acute neurotoxicity. In the MTX cohort, common grade 4 adverse events included neutropenia (19.2%), thrombocytopenia (13.5%), anemia (19.2%), febrile neutropenia/infections (1.9%), mucositis (1.9%), and acute neurotoxicity (1.9%). Common grade 5 adverse events included febrile neutropenia/infections (1.9%), coagulopathy/DVT (1.9%), and acute neurotoxicity (1.9%). In the TEN cohort, the most common grade 4 adverse events included neutropenia (24.2%), thrombocytopenia (9.1%), anemia (3%), and febrile neutropenia/infections (6.1%). Grade 5 febrile neutropenia/infections occurred in only 1 (3%) patient (Table 4).

## Discussion

PCNSL has posed a major challenge to physicians for decades, especially in elderly patients. This study was novel in using TEN as the main agent compared with high-dose MTX in PCNSL. This study showed that PFS and OS were similar in both treatment cohorts. Our study found that the response rate was 90.3% in the MTX cohort and 97.1% in the TEN cohort. No significant differences were observed in median PFS (28.4 months vs 24.3 months) and median OS (31 months vs 32 months) between MTX and TEN cohorts. The results of an open, randomized, phase 2 trial showed that patients with PCNSL treated with methotrexate and methotrexate plus cytarabine had an ORR of 40% and 69%, respectively [5]. A retrospective study found that patients with PCNSL treated with HD-MTX or HD-MTX/rituximab achieved CR rates of 36% and 73% and PFS rates of 4.5 months and 26.7 months, respectively. The median OS was 16.3 months in the HD-MTX cohort and was not yet reached in the HD-MTX/R cohort [23]. In a multicenter single-arm trial, the 2-year PFS was 37.3% and the OS was 47.0% after high-dose methotrexate-based immuno-chemotherapy in elderly patients with PCNSL [3]. Our study was similar to previous studies in terms of PFS and OS, with an advantage in terms of ORR [5, 23, 24]. The TEN-based regimen was non-inferior to the high-dose MTX-based regimen as the first-line therapy for patients with newly diagnosed PCNSL. Our data indicated that TEN was an effective agent in PCNSL. The findings provided another choice for patients with newly diagnosed PCNS instead of high-dose MTX. Although only IELSG risk was associated with OS, radiotherapy as a consolidation therapy improved the PFS.

TEN was used as a main drug in our study for several reasons. It is lipophilic to cross the BB and is eliminated at a slow rate [17]. Several clinical trials showed that primary or metastatic lymphomas of the brain responded to TEN [15, 18, 25]. However, besides TEN, other drugs were also used in most clinical trials, such as fotemustine and methotrexate. Wu et al. [25] reported an ORR rate of 88% in 24 patients treated with teniposide plus fotemustine and dexamethasone, versus 84% in 25 patients treated with high-dose MTX plus cytarabine. In the European Organization for Research and Treatment of Cancer Lymphoma Group phase II trial, 52 patients with PCNSL received teniposide, methotrexate, carmustine, and methylprednisolone combined with radiotherapy; the ORR was 81%, and the 3-year OS was 58% [18]. Therefore, assessing how much TEN contributed to the therapeutic efficacy of PCNSL was extremely difficult. Performing a controlled trial to investigate the efficacy and safety of TEN as the main drug of the regimen compared with high-dose MTX was necessary. In this study, we selected high-dose MTX as the control; the median PFS and OS of patients were 28.4 and 31 months, respectively, consistent with the previous findings [5, 12, 23]. Although no significant differences were found in either ORR or long-term survival rates between both cohorts, our findings suggested that TEN was an effective treatment option for patients with newly diagnosed PCNSL.

Although the combination of HD-MTX-based chemotherapy followed by consolidative WBRT is still commonly used, the role of whole-brain radiotherapy after induction chemotherapy is controversial. G-PCNSL-SG-1 [9], a phase III, randomized, non-inferiority trial showed no statistically significant improvement in PFS with WBRT in patients with newly diagnosed PCNSL who achieved CR after HD-MTX chemotherapy (median PFS 18.3 vs 11.9 months, *P* = 0.14), but omitting WBRT might not be associated with inferior OS (median OS 32.4 vs 37.1 months, HR 1.06). A similar result was obtained in another retrospective research of 103 patients with PCNSL [26]. However, delayed neurotoxicity limited the acceptance of consolidative WBRT as a standard therapy, particularly in patients aged more than 60 years [27]. In a multicenter phase II trial, R-MPV (rituximab, HD-MTX, procarbazine, and vincristine) combined with consolidated reduced-dose WBRT and cytarabine was associated with high response rates, long-term disease control, and minimal neurotoxicity [6]. However, whether chemotherapy or low-dose WBRT contributed to the underlying cause of these results was unclear. To answer this question, the ongoing RTOG 11–14 trial (NCT05011045.) was conducted to explore the impact of consolidative WBRT. In our study, deferred WBRT improved PFS, but not OS. This could be clinically relevant in terms of lower incidence of neurotoxicity.

Whether intravenous rituximab accumulated sufficiently in the CNS to exert an effect was debatable. Holdhoff et al. [23] reported a CR rate of 36% in the HD-MTX cohort and 73% in the HD-MTX/rituximab cohort, with a median PFS of 4.5 months in the HD-MTX cohort and 26.7 months in the HD-MTX/rituximab cohort. The IELSG32 trial showed that the addition of thiotepa and rituximab to high-dose antimetabolites significantly improved ORR, PFS, and OS [28]. However, in a meta-analysis of randomized controlled trials, rituximab in combination with methotrexate-based chemotherapy did not improve OS in immunocompetent patients with newly diagnosed PCNSL [29, 30]. Therefore, the use of rituximab in PCNSL remains a controversial issue, and whether it has a positive impact on patient outcomes remains uncertain. According to the conflicting results in the literature, adding rituximab to treatment protocols of PCNSL (MTX, TEN, MBVP, etc.…) is still debatable. In our study, the addition of rituximab to HD-MTX or teniposide did not cause any significant difference in PFS and OS between the two cohorts of patients.

Nearly half of the patients with PCNSL are aged > 60 years, with 1 tenth of these aged ≥ 80 years [31]. Despite higher methotrexate relative dose intensity (MTX-RDI > 0.75 or not) [32], severe nephrotoxicity caused by MTX cannot be ignored. Whether elderly patients with PCNSL need to receive the same doses of MTX as younger patients remains uncertain [33]. However, no appropriate alternative choice is available for patients with newly diagnosed PCNSL. The data of our study indicated that the toxicity was acceptable and similar between HD-MTX and TEN cohorts. Although no severe renal damage occurred in the HD-MTX cohort, nephrotoxicity remained a concern in the real-life practice.

Our study had a few limitations. It had intrinsic limitations given that it was a retrospective analysis. Also, the radiology review was not done in a blinded manner. Although the survival in the MTX cohort was similar to the published findings [23], the time interval in this study was nearly 10 years, and rituximab was added more recently. Our retrospective analysis included all patients who had received at least 1 cycle of treatment irrespective of their baseline performance status. More than 40% of patients in both cohorts had an ECOG score of 3–4, which was a known prognostic factor in PCNSL [22, 34, 35]. The trial protocol allowed each participating institution to choose whether to administer high-dose chemotherapy followed by ASCT or whole-brain radiotherapy to patients after induction chemotherapy, which might impair comparability between the two cohorts. Finally, as our study is a retrospective, multicenter study, there is an imbalance in the decision of using MTX or teniposide among different research centers due to differences in policies or the experience of clinician.

In conclusion, the findings of this study indicated that TEN was well tolerated and effective in patients with PCNSL and could be used as an alternative to HD-MTX in patients who could not afford the risk of side effects. These findings should be confirmed in a prospective, randomized, controlled trial. In addition, further large-sample studies with longer follow-up are required to investigate the regimen of TEN combined with other drugs.

## Data Availability

All data generated or analysed during this study are included in this published article.

## Abbreviations

ASCT: Autologous hematopoietic stem cell transplantation
BBB: Blood–brain barrier
BSA: Body surface area
CI: Confidence interval
CNS: Central nervous system
CR: Complete response
CSF: Cerebrospinal fluid
DVT: Deep-vein thrombosis
ECOG: Eastern Cooperative Oncology Group
EORTC: European Organization for Research and Treatment of Cancer Lymphoma Group
HD-MTX: High-dose methotrexate
HR: Hazard ratio
IELSG: International Extranodal Lymphoma Study Group
LDH: Lactate dehydrogenase
MTX: Methotrexate
OS: Overall survival
PCNSL: Primary central nervous system lymphoma
PFS: Progression-free survival
PD: Progressive disease
PR: Partial response
SCT: Stem cell transplantation
SD: Stable disease
TEN: Teniposide
UVA: Univariate analyses
WBRT: Whole-brain radiotherapy
